# Tenascin-C expression controls the maturation of articular cartilage in mice

**DOI:** 10.1186/s13104-020-4906-8

**Published:** 2020-02-17

**Authors:** Bastian L. Gruber, Michael J. Mienaltowski, James N. MacLeod, Johannes Schittny, Stephanie Kasper, Martin Flück

**Affiliations:** 1 Laboratory for Muscle Plasticity, Department of Orthopedics, University of Zurich, Balgrist Campus, Lengghalde 5, 8008 Zurich, Switzerland; 2 Gluck Equine Research Center, Department of Veterinary Science, University of Kentucky, Lexington, KY USA; 3 Institute of Anatomy, University of Berne, Berne, Switzerland; 4 Present Address: Department of Animal Science, University of California Davis, Davis, CA USA

**Keywords:** Tenascin C, Knock-out mouse, Articular cartilage, Cell density, Cartilage defect, Load, Adhesion

## Abstract

**Objective:**

Expression of the de-adhesive extracellular matrix protein tenascin-C (TNC) is associated with the early postnatal development of articular cartilage which is both load-dependent and associated with chondrocyte differentiation. We assessed morphological changes in the articular cartilage of TNC deficient mice at postnatal ages of 1, 4 and 8 weeks compared to age-matched wildtype mice.

**Results:**

Cartilage integrity was assessed based on hematoxylin and eosin stained-sections from the tibial bone using a modified Mankin score. Chondrocyte density and cartilage thickness were assessed morphometrically. TNC expression was localized based on immunostaining. At 8 weeks of age, the formed tangential/transitional zone of the articular cartilage was 27% thicker and the density of chondrocytes in the articular cartilage was 55% lower in wildtype than the TNC-deficient mice. TNC protein expression was associated with chondrocytes. No relevant changes were found in mice at 1 and 4 weeks of age. The findings indicate a role of tenascin-C in the post-natal maturation of the extracellular matrix in articular cartilage. This might be a compensatory mechanism to strengthen resilience against mechanical stress.

## Introduction

Tenascin-C (TNC) is a hexameric glycoprotein of the extracellular matrix (ECM) that shapes mechanical and biochemical cues within the cellular microenvironment of various tissues by the modulation of cell adhesion [1]. TNC has a modular composition containing a heptad repeat region, epidermal growth factor (EGF)-like domains, fibronectin-type III repeats, and a fibrinogen-like globe enabling alternatively spliced TNC isoforms to bind different ECM proteins, including syndecan, fibronectin and different integrins [1, 2] and subsequently modify the organization of the cytoskeleton and downstream signalling pathways via the dissolution of focal adhesions [3–5]. This de-adhesive action of TNC allows quiescent cells to enter an intermediate adhesive state that is compatible with tissue remodelling during morphogenesis, wound healing and oncogenic transformation [1, 3, 6, 7].

Expression of TNC is regulated by growth factor- and cytokine-activated signaling pathways [1, 8–10] and is subject to direct and indirect, damage-related regulation by mechanical stress in connective tissue cells [1, 11–13]. Enhanced TNC expression is especially implicated in the adaptive response of musculoskeletal tissues (i.e. skeletal muscle, tendon and bone) to mechanical stress [11–15], which governs the post-natal differentiation, and regenerative response subsequent to the impact of a mechanical challenge or insult, of this tissue family [14, 16–19].

Based on its particularly high abundance in the condensed mesenchyme, TNC has also been implicated in the differentiation of chondrocytes during cartilage maturation in the embryo before TNC expression in these cartilage anlagen is lost and chondrocytes produce cartilage-specific extracellular matrix proteins [20–22]. Later, up to 4 weeks postpartum TNC expression reappears in the peripheral perichondrium [21, 23] and remains expressed in articular cartilage, but not in the growth plate, and decreases thereafter [16, 20, 24]. Recently, TNC has been found to be re-expressed subsequent to traumatic joint loading of the developed articular cartilage and to promote cartilage repair via a switch in extracellular matter synthesis [25].

Although proposed [26], and suggested by TNC’s contribution to musculoskeletal remodelling [11–15] and load-dependent regenerative functional adaptations of joints after birth [27, 28]; it had never been tested experimentally whether TNC participates in articular chondrocyte development and differentiation in long bone models, and remains functional throughout postnatal life. We thus hypothesized that TNC-deficient mice would demonstrate structural aberrations of articular cartilage in the first 2 months after birth when knee joints are first subjected to gravitational loading and chondrocyte volume and extracellular matrix production undergo marked alterations [24].

## Main text

### Methods

#### Study design

TNC-deficient mice (TNC −/−) and homozygous wildtype mice (TNC +/+) were generated by the breeding of homozygous TNC-deficient mice, and homozygous wildtype mice, respectively. Mice were ear marked, genotyped within the first 2 weeks after birth and subsequently housed in groups of 2–6 animals per cage. The parental homozygous TNC-deficient mice and homozygous wildtype mice were derived from the breeding of heterozygous TNC-deficient mice (TNC +/+/). Skeletal tissue was collected from euthanized mice irrespective of sex, at 4 or 8 weeks of age and subjected to histological processing. The assessment of structural deficits (modified Mankin score, cell density in the articular cartilage, TNC expression in articular cartilage) was carried out in a blinded fashion.

#### Animals

TNC-deficient mice were derived from the original strain with a targeted insertion of a β-lactamase cassette in the *Nco*I site of exon 2 of the TNC gene [29] and back crossed with WT 129/SV mice. Mice were housed with 12:12-h light/dark cycle at a constant temperature of 22 °C in Macrolon type III cages (Indulab, Italy) under specific-pathogen-free conditions with standard chow and water ad libitum at the Department of Clinical Research, University of Berne, Switzerland. Animal health status was daily inspected and the microbiological status inspected in sentinels. Genotype was determined by PCR on tail DNA [14]. Tail cutting was done after euthanasia.

#### Sample preparation and histology

The mice were anaesthetized with 5% isoflurane (Provet AG, Burgdorf, Switzerland) and euthanized by decapitation. Skeletal elements of the explanted hindlimbs were fixed in 4% paraformaldehyde and shipped to the University of Kentucky. The tissue was processed by decalcification as described [30, 31], embedded in paraffin, sectioned at 5 μm thickness in parallel direction to the tibial axes, and subjected to standard hematoxylin and eosin (H&E) staining. Slides representing the distal femur from the diaphysis (proximal) to the articular surface of the knee (distal) and the proximal tibia from the articular surface to the diaphysis were shipped to the University of Zurich for morphological analysis.

#### Assessment of structural cartilage deficits

H&E stained sections of the coronary tibia were recorded at a four- and tenfold magnification on an IX50 microscope via a DP72 digital camera (Olympus, Volketswil, Switzerland). A modified Mankin score was used to grade cartilage integrity from 0 to 7 points (i.e. normal structure to complete cartilaginous destruction) based on the staining of the cartilage structure and tidemark (Additional file 1: Figure S1, Additional file 2: Table S1; [32]). The employed scoring rubric has been shown to strongly correlate with the OARSI scoring [33] and has been used consistently in rodent [34, 35] and human specimens to grade mild to moderate cartilage defects [33]. Cell density within the articular cartilage was detected by point counting using a 25 by 25 µm grid that was placed on a randomly selected tenfold magnified microscopic field of each sample under application of the forbidden line rule. Thickness of the tangential/transitional zone of articular cartilage was determined from the average of three measurements for the tangential distance in the center of the joint with the cellSens software (version 1.6, Olympus, Volketswil, Switzerland).

#### Immunohistochemical detection of tenascin-C

Paraffin sections were processed essentially as described [36] but without preincubation with proteolytic enzymes. Deparaffinized sections were incubated with affinity purified TNC specific antibody from rabbit (#473, 1:100; [37]) or a negative control (rabbit antibody against serum response factor, [38]) and subsequently with horse radish peroxidase-coupled goat anti-rabbit antibody [#55676 (1:200; MP Biomedicals, Ohio, USA)]. Signal was detected using AEC high sensitivity substrate (DAKO, Baar, Switzerland) and microscopically recorded.

#### Statistical analysis

We used SPSS by IBM (Armonk, NY, USA) for the statistical analysis and graphic representation of the data. A two-way ANOVA for the factors genotype and age followed by Bonferroni post hoc analysis was performed, when equality of variance could be assumed based on a Levene’s test. A p-value < 0.05 was considered statistically significant.

### Results

#### Animals

All animals entering the experiment were free of signs of stress and had a proper microbiological status. No adverse events were noted.

#### Cartilage structure

**Fig. 1:**
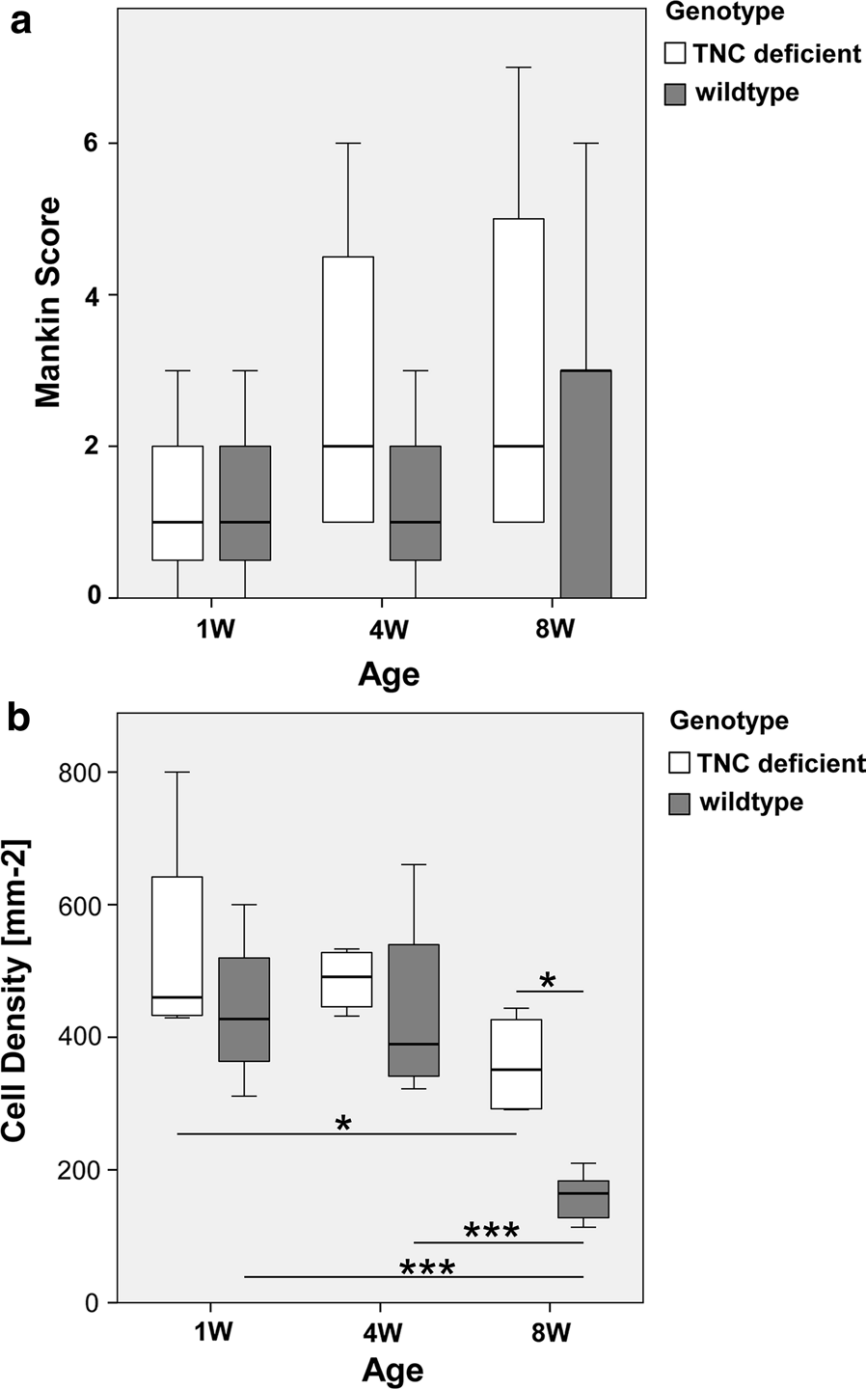
Effect of age and genotype on articular cartilage. Box whisker plots visualizing the median (central line), 25th and 75th percentiles (box), and highest and lowest values (whiskers) for the modified Mankin scores (**a**) and cell density (**b**) in the articular cartilage of wildtype and TNC-deficient mice at 1, 4 and 8 weeks of age. n = 4 for all sample points, except for the 8 weeks of wildtype mice where n = 6. * and *** denote p < 0.05, and < 0.001, respectively, for the indicated difference

Figure 1a shows the results of the assessment using the modified Mankin score. No difference revealed in dependence on genotype (F = 0.701, p = 0.412, η^2^ = 0.034), age (F = 1.223, p = 0.315, η^2^ = 0.109), and the interaction between genotype × age (F = 0.291, p = 0.751, η^2^ = 0.028) (Fig 1).

Development of the tangential/transitional zone of articular cartilage was evident at 4 weeks of age in both wildtype and TNC-deficient mice. An effect of the genotype (F = 9.295, p = 0.010, η^2^ = 0.436) was determined for the thickness of the tangential/transitional zone of articular cartilage. However, thickness was not affected by age (F = 2.677, p = 0.128, η^2^ = 0.182) nor the interaction between genotype × age (F = 0.553, p = 0.471, η^2^ = 0.044). At 8 weeks of age the articular cartilage was thicker in wildtype than TNC-deficient mice (see Table 1).

**Table 1:** Thickness of the tangential/transitional articular cartilage

Thickness (μm)	4 weeks	8 weeks	p-value
WT	125.7 ± 5.3 (4)	140.6 ± 19.8 (5)	0.124
TNC-	107.0 ± 10.9 (4)	108.0 ± 18.7 (4)	0.533
p-value	0.151	0.014	

Median ± standard deviation (n, number of independent replicas) of the average thickness of articular cartilage in wildtype (WT) and TNC-deficient (TNC-) mice at 4 and 8 weeks of age. p-values of the differences are given

#### Altered cell density in articular cartilage of tenascin-C deficient mice

Figure 1b summarizes the measured cell density in the different groups. For cell density in articular cartilage, there was a significant effect of genotype (F = 6.899, p = 0.016, η^2^ = 0.256) and age (F = 11.952, p = 0.001, η^2^ = 0.544), and a trend for an interaction effect of age × genotype (F = 3.494, p = 0.083, η^2^ = 0.200). Post hoc analysis localized a significant difference (p = 0.011) between the TNC-deficient (359.4 ± 54.5 cells/mm^2^) and wildtype (160.8 ± 44.5 cells/mm^2^) mice at 8 weeks of age. Cell density between TNC-deficient and wildtype mice at one (p = 0.229) and 4 weeks (p = 0.544) of age did not differ significantly. In TNC-deficient mice cell density was lower at eight than 1 week of age (− 178.8 cells/mm^2^, p = 0.031). In wildtype mice, cell density was lower at eight than 1 week of age (− 281.0 cells/mm^2^, p = 0.001) and 4 weeks of age (− 279.7 cells/mm^2^, p = 0.001). The diameter of the epiphysis demonstrated an effect of age (F = 54.156, p < 8.5 10^−9^, η^2^ = 0.844), but no effect of genotype (F = 0.004, p = 0.951, η^2^ = 0.001), increasing similarly between one and 4 weeks of age in both genotypes and then remained stable (Additional file 3: Figure S2).

#### Localization of tenascin-C expression

**Fig. 2:**
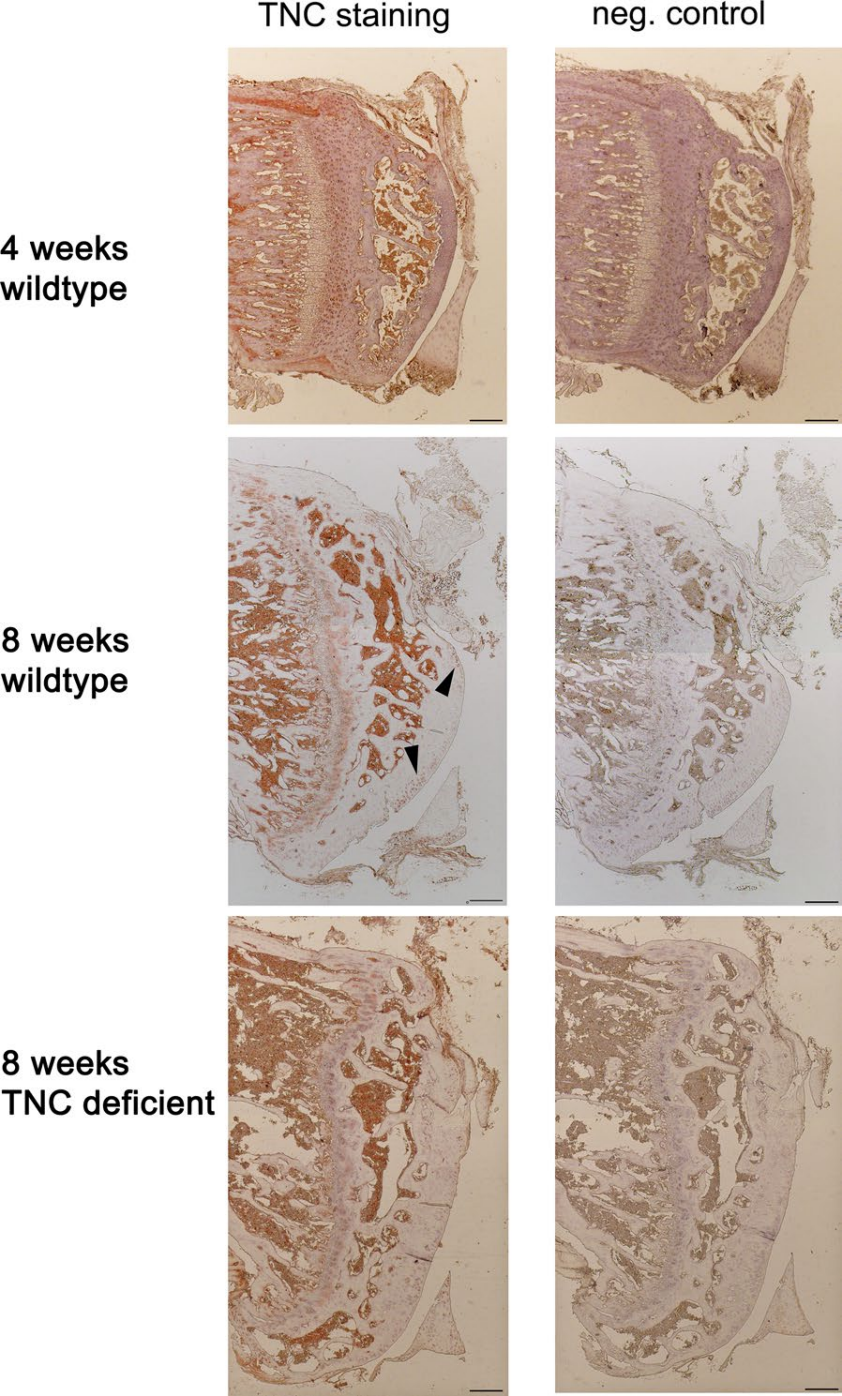
Tenascin-C expression in articular cartilage. TNC signal in a 4 and 8 weeks old wildtype mouse and an 8 weeks old TNC-deficient mouse. The detected signal after detection with TNC antibody was compared to a negative control. Arrowheads point to TNC positive staining in association with chondrocytes. Bar 200 μm

Chondrocyte specific staining of TNC was identified in the tangential and transitional zone of the tibial cartilage in 4 and 8 weeks-old wildtype and TNC mice and in the bone marrow (Fig. 2; Additional file 4: Figure S3).

### Discussion

Our study has examined the articular cartilage of TNC-deficient mice at early age. The results indicate that TNC-deficient mice demonstrate alterations in the maturation of the tibial articular cartilage at 8 weeks of age that do not seem to manifest in a gross pathology according to Mankin scores under native conditions. Our data imply that the reduction in chondrocyte cell density that characterizes post-natal growth and maturation of skeletal development [24, 30, 39] is TNC-dependent.

Our immunohistochemical experiments localizing strong TNC expression to chondrocytes of the tangential/transitional zones of 4- and 8-weeks-old mice confirm the association of TNC expression with the periphery of developing cartilage [16, 36]. This result indicates that chondrocyte-related TNC expression and differences in the density of this cell type in articular cartilage 8 weeks into development are associated. TNC expression is abundant in the territorial matrix of cartilage [16, 36] suggesting its involvement in the genesis of articular chondroytes and assembly of the chondrocyte matrix [21, 36]. Our observations imply that TNC is part of the mechanism regulating the increase in volume of articular cartilage, and extracellular matrix production from birth to 2 months of age in mice, when overall cell density is decreased [24]. Effects of TNC-deficiency on the thickness of cell layers had been documented during healing of compressed corneas [40] and trauma induced extracellular matrix synthesis in articular cartilage [25, 36]. In this regard, the reduced thickness of articular cartilage in TNC-deficient mice at 8 weeks of age suggests a reduced capacity for extracellular matrix synthesis.

We observed that the tangential/transitional zone of articular cartilage was 30% thicker in wildtype than TNC-deficient mice at the age of 8 weeks when chondrocyte density in the wildtype mice was concurrently lower. The findings imply that the lower chondrocyte density in wildtype mice is in part explained by the enhanced deposition of extracellular matrix rather than a genotype effect on the proliferation or depletion of chondrocytes [41]. This contention is supported by the reciprocal relationship between the expression of TNC and cartilage-specific extracellular matrix proteins during chondrocyte differentiation [21], and between the cell density and thickness of articular cartilage [40]. Meanwhile, the decreased cell density in articular cartilage of wildtype mice between 4 and 8 weeks of age, when the thickness of articular cartilage was not significantly affected (12%, p = 0.124), suggests that cells were nevertheless lost during this phase of postnatal development; contributing via a reduction in the capacity for extracellular matrix synthesis [22], to the load-dependent thinning of articular cartilage during subsequent post-natal development [27].

We conclude that the morphological changes in the articular cartilage of TNC-deficient mice are subtle. They do not have a higher rate of growth abnormalities or structural defects; however, structural differences at 8 weeks of age are supportive of a TNC-dependent mechanism that leads to the deposition of the territorial matrix, rather than the down-regulation of chondrocyte density, in the 1 weeks after birth [19].

### Limitations

Our histological assessment does with the exception of TNC expression only allow limited conclusions on the molecular pathways being implicated in the observed differences in post-natal development of articular cartilage. In this regard we also refer to the observation that the strain of TNC-deficient mice retains expression of an aberrant form of TNC that may lead to intracellular immunoreactivity [14, 42]; in line with the observed TNC staining in the cytoplasm of chondrocytes (Fig. 2). Moreover, we only observed a relatively small sample size of 26 animals during the first 8 weeks of life. This is possibly too short a time period to observe the TNC-dependent repair mechanisms [43], as cartilage repair is comparably slow and sub-optimal [26, 44].

## Supplementary information

**Additional file 1: Figure S1.** Articular cartilage of a TNC deficient mouse at 8 week of age. The articular cartilage is divided into 4 zones as depicted. The tidemark lies between the radial and the calcified zone.

**Additional file 2: Table S1.** List of the discriminative characteristics for the two considered structural aspects which respective points are summed to reveal the modified Mankin score.

**Additional file 3: Figure S2.** Box Whisker plots visualizing the median (central line), 25th and 75th percentiles (box), and highest and lowest values (whiskers) for the diameter of the epiphysis in wildtype and TNC-deficient mice at 1, 4 and 8 week of age. n = 4 for all sample point, except for all sample points, except for the 8 week of wildtype mice where n = 6.***denotes p < 0.001 for the indicated difference.

**Additional file 4: Figure S3.** Tenascin-C expression in articular cartilage. TNC signal in articular cartilage of an 8-week-old wildtype and TNC-deficient mouse. The detected signal was compared to a negative control. Arrowhead points to TNC positive staining in association with chondrocytes. Bar, 200 μm.

## Data Availability

The dataset supporting the conclusions of this article is available in the Mendeley data repository, (https://data.mendeley.com/) under 10.17632/mt3txxw53g.1.

## Abbreviations

Akt: Protein kinase B
CREB: cAMP-responsive element binding protein
ECM: Extracellular matrix
EGF: Epidermal growth factor
H&E: Hematoxylin and eosin
MAP: Mitogen activated protein
PDGF: Platelet-derived growth factor
PI3K: Phosphoinositide 3-kinase
TGFβ: Transforming growth factor beta
TNC: Tenascin-C
WT: Wildtype
