# Supplementary figures and images for: Tenascin-C expression controls the maturation of articular cartilage in mice
Source: BMC Res Notes. 2020 Feb 17;13:78. doi: 10.1186/s13104-020-4906-8 (PMC7027060; doi:10.1186/s13104-020-4906-8)

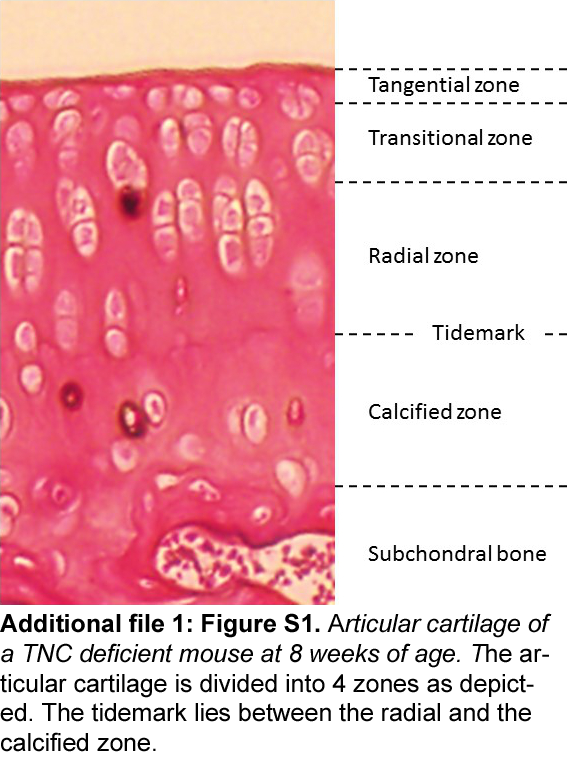

Supplement: Supplementary file 1 — Additional file 1: Figure S1. Articular cartilage of a TNC deficient mouse at 8 week of age. The articular cartilage is divided into 4 zones as depicted. The tidemark lies between the radial and the calcified zone. [file 13104_2020_4906_MOESM1_ESM.tif]

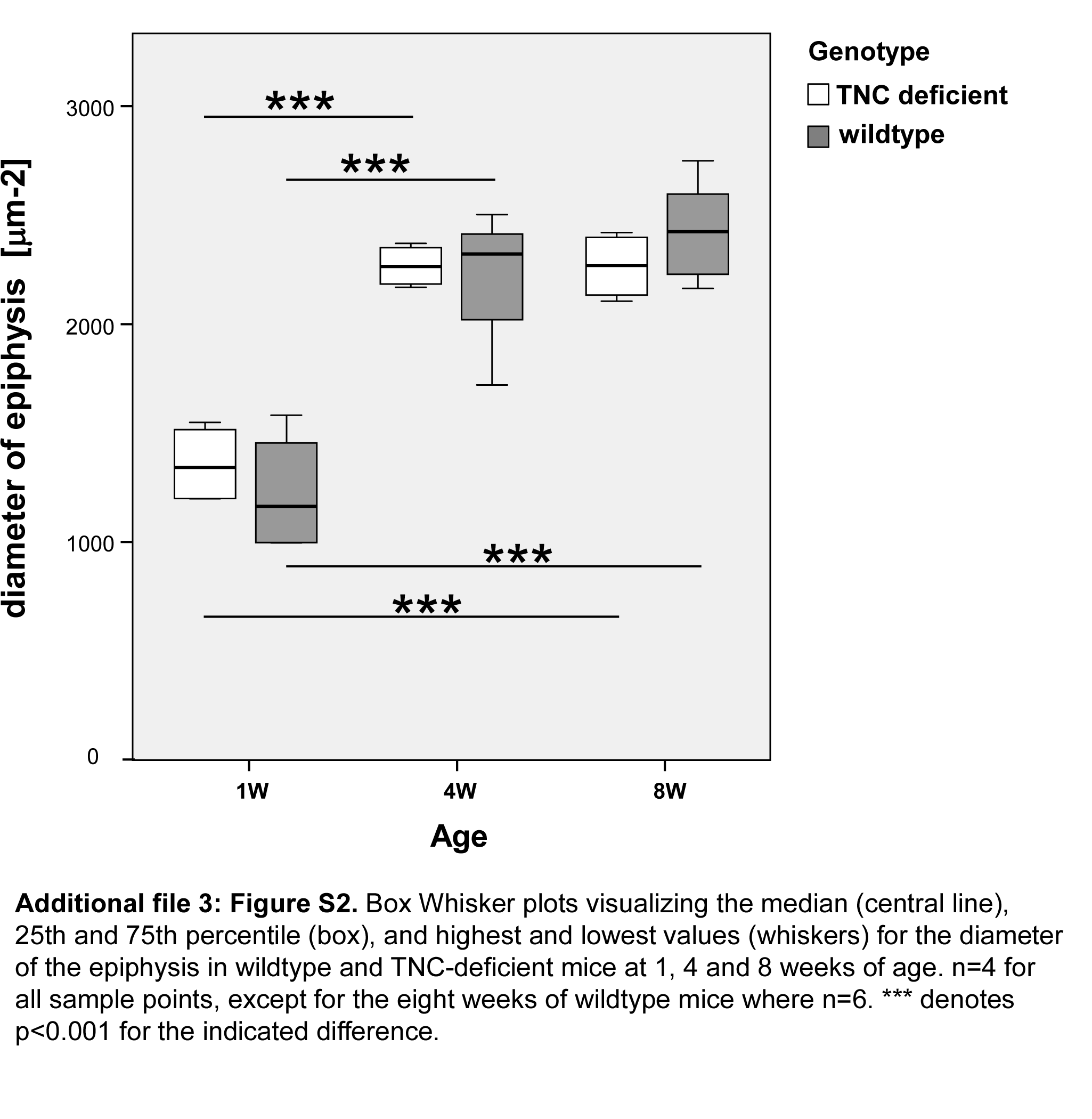

Supplement: Supplementary file 3 — Additional file 3: Figure S2. Box Whisker plots visualizing the median (central line), 25th and 75th percentiles (box), and highest and lowest values (whiskers) for the diameter of the epiphysis in wildtype and TNC-deficient mice at 1, 4 and 8 week of age. n = 4 for all sample point, except for all sample points, except for the 8 week of wildtype mice where n = 6.***denotes p < 0.001 for the indicated difference. [file 13104_2020_4906_MOESM3_ESM.tif]

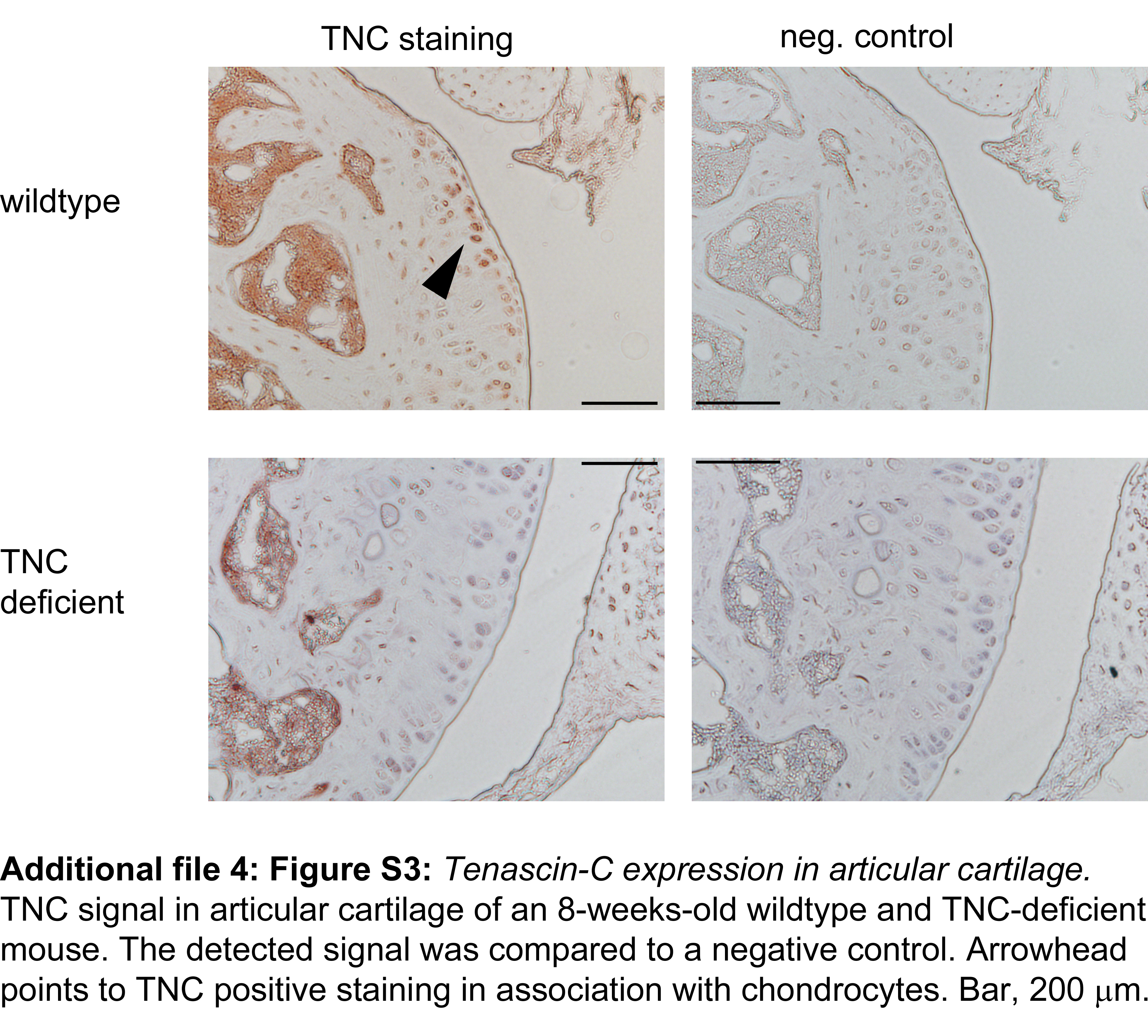

Supplement: Supplementary file 4 — Additional file 4: Figure S3. Tenascin-C expression in articular cartilage. TNC signal in articular cartilage of an 8-week-old wildtype and TNC-deficient mouse. The detected signal was compared to a negative control. Arrowhead points to TNC positive staining in association with chondrocytes. Bar, 200 μm. [file 13104_2020_4906_MOESM4_ESM.tif]
