# Supplementary material for: Tenascin-C expression controls the maturation of articular cartilage in mice
Source: BMC Res Notes. 2020 Feb 17;13:78. doi: 10.1186/s13104-020-4906-8 (PMC7027060; doi:10.1186/s13104-020-4906-8)
Supplement: Supplementary file 2 — Additional file 2: Table S1. List of the discriminative characteristics for the two considered structural aspects which respective points are summed to reveal the modified Mankin score. [file 13104_2020_4906_MOESM2_ESM.doc]

**Additional file 2: Table S1.** List of the discriminative characteristics for the two considered structural aspects which respective points are summed to reveal the modified Mankin score.

*Characteristics points*

**Articular cartilage structure** Normal 0

surface irregularities 1

Pannus 2

cleft to transitional zone 3

cleft to radial zone 4

cleft to calcified zone 5

total disorganization 6

**Tidemark** Intact 0

disrupted 1
